# Data Linkage in Australia: The First 50 Years

**DOI:** 10.3390/ijerph182111339

**Published:** 2021-10-28

**Authors:** Merran Smith, Felicity Flack

**Affiliations:** Population Health Research Network, University of Western Australia, 35 Stirling Highway, Crawley 6009, Australia; felicity.flack@uwa.edu.au

**Keywords:** data linkage, Australia, cross-jurisdiction

## Abstract

Population-based data linkage has a long history in Australia from its beginnings in Western Australia in the 1970s to the coordinated national data linkage infrastructure that exists today. This article describes the journey from an idea to a national data linkage network which has impacts on the health and well-being of Australians from preventing developmental anomalies to responding to the COVID-19 pandemic. Many enthusiastic and dedicated people have contributed to Australia’s data linkage capability over the last 50 years. They have managed to overcome a number of challenges including gaining stakeholder and community support; navigating complex legal and ethical environments; establishing cross-jurisdictional collaborations, and gaining ongoing financial support. The future is bright for linked data in Australia as the infrastructure built over the last 50 years provides a firm foundation for further expansion and development, ensuring that Australia’s linked health and human services data continues to be available to address the evolving challenges of the next half century.

## 1. Introduction

Data linkage is a method of bringing together information derived from different sources, but relating to the same individual or event in a single file [1]. It is not a new method; in fact, it predates the introduction of modern computers [1]. For example, in the late 18th century, Edward Jenner conducted what is thought to be the first data linkage, which provided evidence of the efficacy of smallpox vaccination [2].

The term “record linkage” first appeared in the literature in the 20th century in an article by Dr Halbert Dunn, the head of the United States National Office of Vital Statistics, in 1946. In this article, Dunn provides an eloquent description of data linkage and the value of linked data systems.

“Each person in the world creates a Book of Life. This Book starts with birth and ends with death. Its pages are made up of the records of the principal events in life. Record linkage is the name given to the process of assembling the pages of this Book into a volume” [3].

The advent of more advanced mathematical matching techniques and computer technology has enabled the expansion of the use of linked data for population-wide research. It has enabled research across populations and across the life course. Data linkage has become an essential tool in the ongoing understanding and improvement of health and social services worldwide. Linked data is used to:

- Assess outcomes of clinical or therapeutic interventions;
- Assess the safety, quality and costs of health care and other government services;
- Explore the relationships between personal, economic, environmental and lifestyle factors;
- Investigate social and community influences on individual and community health and well-being;
- Assess the effectiveness of preventative programs;
- Obtain valuable follow-up information on participants in research studies and surveys [4].

Whilst the benefits of data linkage can be clearly demonstrated, there are also risks. Linking data and the analysis of linked data requires the use of person-level data often without informed consent. This means that data linkage operates within complex legal and ethical frameworks designed to protect and balance the multiple interests at play. The history of data linkage is infused with the challenges associated with balancing the benefits against the risks to individuals and groups.

Australia has been at the forefront of the uses of linked data and development of linkage systems for more than fifty years. It is timely to reflect on the progress and achievements of the last half century and consider the future of linked data in Australia. This paper outlines Australia’s data linkage journey, from its beginning in the 1970s up to the present day. Current challenges and plans for future development are also discussed.

## 2. The Early Years: Ad Hoc and Other Linkages (1970–1994)

In 1967, Professor Michael Hobbs returned to Western Australia from Oxford University, where he had been involved with the Oxford Record Linkage Study [5]. In 1970, he published a plan for the introduction of medical record linkage studies in Australia [1]. The plan included the initial linkage of birth, marriage and death records, census data, perinatal mortality and hospital morbidity records. This could be subsequently supplemented by:

“Records of physical or mental disability

Records of exposure to new industrial processes

Prescriptions of selected drugs

Notifications of infectious disease.” [1]

Additional elements of the plan were described as follows:

“A plan for the introduction of medical record linkage studies in Australia on a National or State-wide basis must therefore include the following:

1. The interest and co-operation of the Bureau of Census and Statistics.
2. Preferably the introduction of a unique numbering system, but failing this, the collection of uniform identifying data on all records for which linkage is planned, either routinely or on an ad hoc basis.
3. The agreement by holders of important data to the release of information for linkage procedures under the auspices of the Bureau of Census and Statistics.
4. The realization by users of such data that tabulations identifying individual persons will not be practicable.
5. The awakening of interest in, and realization of the uses of, record linkage in Government Departments, Medical Administrators and research workers.
6. The implementation of a pilot record linkage scheme in Western Australia.” [1]

This plan was the beginning of an Australian national linkage system which 51 years later incorporates many elements of the original plan. However, in 1970, systematic approaches to data linkage at the state or national level were still a long way off. Professor Hobbs and three other Western Australian researchers, Professor Bruce Armstrong, Professor Fiona Stanley and Professor D’Arcy Holman, were instrumental over the next 25 years in achieving a systematic data linkage system in Western Australia [1,5,6,7].

Western Australia was well positioned to have a population-based data linkage system as it was the only state to have implemented a state-wide hospital reporting system. From the mid-1970s, this included the standardised collection of names, which enabled high-quality linkage. Other elements that proved essential to the establishment of the Western Australian Data Linkage System were researcher champions including Professors Hobbs, Stanley and Holman, who understood the value of linked data, had the persistence to obtain it and the skills to use it, as well as strong collaboration between the Western Australian Department of Health and the University of Western Australia [6].

In 1977, Professor Stanley’s research group established the Western Australian Maternal and Child Health Research Database, which was later housed at the Institute for Child Health Research (now Telethon Kids Institute). This was a linked database containing the following state-wide data collections:

- Midwives notifications,
- Birth records,
- Death records,
- Hospital morbidity,
- The WA Birth Defects Registry (now the WA Register of Developmental Anomalies), and
- The Cerebral Palsy Register.

This unique linked data collection was an extremely valuable resource for a wide range of maternal and child health research, particularly the causes of stillbirth and risks factors for cerebral palsy [8]. The types of research supported included:

- Descriptive epidemiology of perinatal and paediatric outcomes [9],
- Case control studies [10],
- Cohort studies [11], and
- Studies to evaluate care [12].

During this time, Professors Stanley and Bower conducted their world leading research on the link between folate deficiency and neural tube defects [13]. Later, they went on to demonstrate that folate supplementation and fortification prevent these defects, a major contribution to public health [14].

## 3. The Introduction of State-Based Systematic Data Linkage: The Western Australian Data Linkage Branch and New South Wales Centre for Health Record Linkage (1995–2008)

### 3.1. Establishment and Development of the Western Australian Data Linkage Branch

In 1994–1995, Professor Holman was appointed as the inaugural Chair in Public Health at the University of Western Australia. Professors Holman, Hobbs and others put in a successful application to the Western Australian Lotteries Commission to establish a Health Services Research Linked Database [5]. The three-year grant enabled establishment of a data linkage unit within the Western Australian Department of Health. The initiative was supported by Professor Ian Rouse, the Department of Health’s Director of Health Statistics and Dr John Bass was the first linker. Initial work was focused on creating probabilistic linkages within and between six core data collections, including births, deaths, hospital separations, midwives notification and cancer registry data [15,16].

The Lotteries Commission grant concluded in 1997–1998 and Dr Merran Smith, the then Director of the Department of Health’s Heath Information Centre, submitted a successful proposal to establish data linkage as a core Departmental service. Additional health data collections were added to the data linkage system and additional staff were taken on to meet the growing demand for linked data. In 1999, the Department of Health became the principal funder and the Western Australian Data Linkage Branch which incorporated the data linkage unit was established within the Health Information Centre. The Western Australian Data Linkage System was one of only five such comprehensive systems in the world at the time [16]. Further information about the Western Australian Data Linkage Branch is available at https://www.datalinkage-wa.org.au/ (accessed on 21 October 2021).

A Management Committee was subsequently established to oversee the Western Australian linked data resources. This included representatives of the Department of Health, the University of Western Australia and the Institute for Child Health Research.

In 2002, an initiative commenced to create intergenerational family linkages (the Family Connections project) [17]. Data from other Western Australian Government agencies was also incorporated into the Western Australian Data Linkage System, including data from Education, Community Services and Justice Departments. Some data from these agencies was subsequently housed in a Custodian Controlled Research Extracts Server (CARES) to facilitate supply of linked data for approved projects [18].

### 3.2. Establishment of the Centre for Health Record Linkage (CHeReL)

In 1994, the then New South Wales Department of Health established a record linkage service to support health research and management of health services. In 2005, following marked increases in the demand for linked data, the Sax Institute commissioned Professor Holman and others from Western Australia to develop a case for a data linkage facility in New South Wales and to recommend a preferred model based on international best practice and stakeholders views. In 2006, eight organisations including New South Wales Health, the Australian Capital Territory Department of Health, Cancer Institute New South Wales and the Sax Institute, agreed to contribute funding for the first three years of operation of the CHeReL. The CHeReL subsequently transitioned to a business unit of New South Wales Health, primarily funded by the New South Wales Ministry of Health. It is of note that the CHeReL was established to undertake data linkage for both New South Wales and the Australian Capital Territory and this arrangement is continuing [19]. Further information about the CHeReL is available at https://www.cherel.org.au/ (accessed on 21 October 2021).

### 3.3. Challenges and Benefits

Challenges to establishing the Western Australian and New South Wales/Australian Capital Territory data linkage systems included support from decision-makers to establish the systems. For both the Western Australian Data Linkage System and the CHeReL, support from research users was a significant factor with each starting out as a collaboration between research groups and government agencies. It is of note that both units moved to a majority government agency support within a few years of establishment and both are now located within their respective state government health agency.

The Western Australia and New South Wales/Australian Capital Territory initiatives demonstrated the benefit of routinely updated, population-based, data linkage systems rather than ad hoc, project-based data linkages. Once established, there were many other benefits including cheaper projects, better data and improved privacy protection [15]. However, the time needed to obtain project approvals was often lengthy, especially for complex projects, and there were delays in supply of linked data. There were also challenges with linkage of data between Australian jurisdictions.

Privacy and confidentiality concerns from data custodians and the community were always a high priority and shaped the approach to systematic linkage in Western Australia and New South Wales. The use of the “best practice protocol”, which requires the separation of identifiers from health data as well as the separation of roles, i.e., the people with access to the identifiers do not have access to the health data, was central to addressing these concerns [20].

In addition, strong community support for data linkage activities is essential to running a successful data linkage system. Both these data linkage systems have incorporated community involvement activities in their development and operations. In Western Australia, the Consumer and Community Health Research Network established in 1998 by the School of Population Health at the University of Western Australia has been particularly influential and supportive [21].

Moral and ethical issues around balancing all the interests at play, not just privacy, also had to be tackled. These issues included the protection of government interests and the possibility that not using linked data could result in harms by preventing or delaying health and health service improvements [22,23,24].

### 3.4. Early Cross-Jurisdictional Linkage

Australia is a federation with a complex health system. The Australian Government is responsible for some aspects of this system, with state and territory governments responsible for other aspects. As a result, some population health data is held by Australian Government agencies while other data is separately held by government agencies in six states and two territories.

One of the first examples of cross-jurisdiction data linkage in Australia was the Australian Cancer Statistics Clearing House. This was established in 1986 at the Australian Institute of Health and Welfare (AIHW). It linked cancer registry and death data from cancer registries in Australian states and territories to provide national cancer information. However, this data was not routinely linked to other population health data.

In 1998, discussions commenced to enable linkage of Australian Government Medical Benefits Schedule (MBS) payments and Pharmaceutic Benefits Scheme (PBS) data to the Western Australian linked hospitalisations and death data for a project on diabetes. A Memorandum of Understanding (MoU) to cover the linkage was developed between the (then) Commonwealth Department of Health and Aged Care, the Health Department of Western Australia, the University of Western Australia, the AIHW and the Health Insurance Commission. The MoU was signed in 2001 with particular support from the Secretary of Commonwealth Health (Mr Andrew Podger), the Commissioner of the Health Department of Western Australian (Mr Alan Bansemer) and the Director of AIHW (Dr Richard Madden) and the linkages were undertaken [20]. In 2003, the arrangement was expanded to establish a population-based, cross-jurisdiction linked data resource. The arrangement was supported by a cross-jurisdiction management committee which also considered applications for access to the linked data. It was the first time that this type of population-based, cross-jurisdiction data linkage resource was available in Australia. It enabled important research projects on topics such as potentially inappropriate medications in the elderly and the impact of regular primary care on outcomes of people with chronic diseases to be completed [25,26,27,28].

### 3.5. Challenges and Achievements

Challenges in establishing and accessing the cross-jurisdiction linked data resource included complex legal frameworks, lengthy project approval processes and delays in data supply. Changes in personnel in participating agencies also had an impact, with new appointees not always as supportive of cross-jurisdiction data linkage as their predecessors. The resource was last updated in 2007 and subsequently discontinued. Each of the challenges identified through the cross-jurisdiction data linkage project was complex in its own right and none was amenable to a simple solution. These challenges informed plans for the development of a more coordinated Australian data linkage system (see Section 4 below).

Although the resource was discontinued, the work clearly demonstrated the feasibility of population-based linkage of Australian Government and state government data, at both the governance and technical levels. It also highlighted the importance of access to cross-jurisdiction linked data in the Australian context.

## 4. National and Cross-Jurisdictional Data Linkage: The Population Health Research Network (PHRN) (2009–2021)

### 4.1. Establishment of the PHRN

In its 2004–05 Budget, the Australian Government announced the implementation of the National Collaborative Research Infrastructure Strategy (NCRIS) with the objective of bringing more strategic direction to the investment in national research infrastructure. This was followed in 2006 by the first NCRIS Strategic Roadmap. The Roadmap was developed after extensive national consultation and set the priorities for the Australian Government’s investment in national research infrastructure.

The 2006 Roadmap proposed the scoping of a coordinated national data linkage capability.

“One possibility is that the capability could be modelled on the system that is being implemented in Western Australia …. A national system might comprise a network of such data linkage units with oversight by a coordination authority provided with both funding and staff capable of providing both intellectual leadership and administrative support” [29].

A lengthy national scoping and consultation period followed, with the final investment plan for a national data linkage capability accepted in 2009. The investment plan identified the University of Western Australia as lead agent for the PHRN and covered the establishment of a central coordinating office at the University, four new state/territory data linkage units based on the Western Australian model to complement the existing units in Western Australia and New South Wales, a national data linkage unit and a secure, remote access, data laboratory. The investment plan was designed to deal with the challenges of the complex Australian health system, to improve health and well-being, and enhance the effectiveness and efficiency of health services.

Contracts were signed and the establishment of PHRN commenced in April 2009 with the appointment of the Chief Executive. A detailed description of the PHRN data linkage infrastructure was published in 2019 [30]. Further information about PHRN is available at https://www.phrn.org.au/ (accessed on 21 October 2021).

Australia is a federation of six states and two self-governing territories which together make up the Commonwealth of Australia. State and territory governments are responsible for some aspects of health care, while the Australian Government is responsible for other aspects. The networked and coordinated approach to a national data linkage system implemented by the PHRN is a response to the unique characteristics of the federation and the Australian health system. This approach has similarities to the Health Data Research Network Canada which is working towards harmonizing data and linkage systems across many provinces [31]. Other smaller single jurisdiction nations such as New Zealand and Denmark have implemented centralized linkage systems, an approach which was not feasible in Australia [32,33].

### 4.2. Challenges and Achievements

The establishment of a national, coordinated data linkage infrastructure across nine jurisdictions should not be underestimated. While a distributed, federated system was the agreed approach, it also posed a number of challenges including:

- Standardisation of data and metadata across jurisdictions;
- Standardisation or benchmarking of linkage methods;
- Standardising, harmonising or coordinating approval requirements and processes;
- Different legislation, regulation, policy and culture between jurisdictions;
- Varying levels of data linkage experience and expertise between jurisdictions.

Another challenge was to get all jurisdictions to participate. While a majority of jurisdictions participated from the outset, it is only since 2011 that all Australian jurisdictions have participated.

Being a truly national network that links data from all Australian, state and territory governments was a priority for the PHRN from the beginning. Achieving participation from all jurisdictions meant supporting a high level of flexibility in how the data linkage infrastructure was developed, implemented and operated in each jurisdiction. Strict requirements for each jurisdiction to implement and operate in specific nationally agreed ways would have delayed the participation of some jurisdictions and it is possible that a national network of any kind may not have been achieved. However, the distributed approach resulted in differences in data linked, linkage methods and approval processes across jurisdictions, which make multi- and cross-jurisdictional research projects more complex than they would be with a standardised national approach [34,35].

To help address these challenges, the PHRN holds regular meetings with senior officers in participating organisations and hosts regular forums for technical staff. It is also working with jurisdictions to establish enduring cross-jurisdiction data linkage and related linked data assets.

A further challenge has been Australia’s complex authorising environment, with each jurisdiction having its own set of enabling legislation and related policies and practice. PHRN continues to participate in jurisdictional and national processes aimed at simplifying the authorising environment. One success was the establishment of mutual acceptance of ethical review for data linkage projects. The PHRN also established and continues to operate an Online Application System and related services to assist researcher access to cross-jurisdiction linked data. There is strong growth in demand for this service [36].

The PHRN’s most significant achievement is the establishment of a coordinated national system of cross-jurisdiction linkage. This system is internationally unique and enables health and human services data from different jurisdictions about the same individual to be linked and accessed. All Australian jurisdictions now have at least 10 years of their core population health data linked. Over 210 data collections are routinely linked and there are more than 15 billion records in the national linkage system. There is also regular linkage to national clinical registries and large longitudinal cohorts. In addition, Australian linked data is being increasingly used for clinical trials. There has been a steady increase in the number of data applications and peer reviewed publications using linked data since the establishment of the PHRN [36,37,38].

PHRN also pioneered national secure remote access data laboratories in Australia. The first, the Secure Unified Research Environment at the Sax Institute in Sydney, is now a well-used environment trusted by custodians across Australia to provide access to sensitive unit record data [36,38].

Research using PHRN linked data has positively impacted many aspects of health and other human services across Australia. This includes changes to government policy as well as changes in clinical practice. Information on impacts is available on the PHRN website [39]. One important example relates to the introduction of Human Papilloma Virus (HPV) vaccination to prevent cervical cancer. The vaccine was introduced in Australia in 2007 and monitored by linking vaccine registers to cervical smear registers in two states (Queensland and Victoria) [40,41,42]. Findings from these and related studies resulted in a change to Australia’s cervical cancer screening program, with cytology screening every two years replaced with more accurate HPV screening every five years. The current COVID-19 pandemic is a further example, with Australia’s linked data playing a significant part in jurisdiction responses to the pandemic [43].

While there has been an expansion of population-based data linkage capability across the globe in recent years, Australia remains one of a relatively small number of countries with a national population-based data linkage capability.

## 5. The Future of Data Linkage in Australia

The demand for access to high-quality linked data from an ever-expanding range of sources (omics, environment, clinical records, wearable devices, social media, etc.) is likely to accelerate over coming years. This demand will come from a wide range of users including governments, academia and private industry.

Close collaboration will be required across Australian jurisdictions, the research sector and industry to meet this demand, including to source the data required, routinely link across jurisdictions and provide access to linked data in efficient and safe ways. Changes to Australia’s very complex authorising environment may also be needed to ensure that the rights and interests of stakeholders are carefully considered and the approval and access processes are proportionate to the risks as systems evolve. New technical advances in computing infrastructure and analytical techniques will make data linkage and the analysis and management of linked data more accurate, efficient and safe. New approaches to metadata and data standardisation for huge volumes of data from very different sources will also be required to enable people to find suitable data, and plan and execute their research.

In addition, it will be necessary to ensure that the Australian community supports and trusts the data linkage system and that linked data is used in ways that demonstrate clear public benefit. The community will need to become more data literate and better understand both the benefits that linked data can bring and the risks to individuals and groups. It may not be possible to ensure anonymity given the volume of data on each individual and the emerging analytical tools. A multi-pronged approach to community education and involvement will be required including:

- The provision of information about the benefits, risks and risk mitigation strategies on a range of communication platforms,
- Community involvement in setting the research agenda, and
- Community representation on decision making and advisory groups.

## 6. Conclusions

The first 50 years of data linkage development in Australia has provided a firm foundation for further expansion and development, and will help to ensure that Australia’s linked health and human services data continues to be available to address the evolving challenges of the next half century.

## Data Availability

Not applicable.

## References

[1] Hobbs M., McCall M. (1970). Health statistics and record linkage in Australia. J. Chronic Dis..

[2] Machado C.J., Hill K. (2004). Probabilistic record linkage and an automated procedure to minimize the undecided-matched pair problem. Cad Saude Publica.

[3] Dunn H.L. (1946). Record linkage. Am. J. Public Health.

[4] Adams C., Flack F., Allen J. Research using linked data. Sharing Linked Data for Health Research: Toward Better Decision Making.

[5] Hobbs M. Michael Hobbs Interview 24 January 2013. https://oralhistories.arts.uwa.edu.au/items/show/45.

[6] Armstrong B.K., Kricker A. (1999). Record linkage—A vision renewed. Aust. N. Z. J. Public Health.

[7] The School of Population Health Welcomes the Return of Professor Bruce Armstrong as Adjunct Professor. https://www.news.uwa.edu.au/archive/201512228287/appointments/school-population-health-welcomes-return-professor-bruce-armstrong-adjunct/.

[8] Stanley F.J., Croft M.L., Gibbins J., Read A.W. (1994). A population database for maternal and child health research in Western Australia using record linkage. Paediatr. Perinat. Epidemiol..

[9] Stanley F.J., Hobbs M.S. (1981). Perinatal outcome in Western Australia, 1968 to 1975 3. Causes of stillbirths and neonatal deaths excluding congenital malformations. Med. J. Aust..

[10] Alessandri L.M., Stanley F.J., Garner J.B., Newnham J., Walters B.N.J. (1992). A case-control study of unexplained antepartum stillbirths. BJOG Int. J. Obstet. Gynaecol..

[11] Stanley F.J., English D.R. (1986). Prevalence of and risk factors for cerebral palsy in a total population cohort of low-birthweight (less than 2000g) infants. Dev. Med. Child Neurol..

[12] Stanley F.J., Hobbs M.S.T. (1980). Neonatal mortality and cerebral palsy: The impact of neonatal intensive care. J. Paediatr. Child Health.

[13] Bower C., Stanley F.J. (1989). Dietary folate as a risk factor for neural-tube defects: Evidence from a case-control study in Western Australia. Med. J. Aust..

[14] Bower C., Ryan A., Rudy E., Miller M. (2002). Trends in neural tube defects in Western Australia. Aust. N. Z. J. Public Health.

[15] Holman C.D., Bass A.J., Rosman D.L., Smith M.B., Semmens J.B., Glasson E.J., Brook E.L., Trutwein B., Rouse I.L., Watson C.R. (2008). A decade of data linkage in Western Australia: Strategic design, applications and benefits of the WA data linkage system. Aust. Health Rev..

[16] Holman C.D. (2014). 20 Years of Discovery and Advance 1994–5 to 2013–14.

[17] Glasson E.J., de Klerk N.H., Bass A.J., Rosman D.L., Palmer L.J., Holman C.D.J. (2008). Cohort profile: The Western Australian family connections genealogical project. Int. J. Epidemiol..

[18] Eitelhuber T., Davis G., Rosman D.L., Glauert R. (2014). Western Australia unveils advances in linked data delivery systems. Aust. N. Z. J. Public Health.

[19] Irvine K., Hall R., Taylor L. (2019). Centre for Health Record Linkage: Expanding access to linked population data for NSW and the ACT, Australia. Int. J. Popul. Data Sci..

[20] Kelman C., Bass A., Holman C. (2002). Research use of linked health data-a best practice protocol. Aust. N. Z. J. Public Health.

[21] Consumer and Community Involvement Program. About Us. https://cciprogram.org/about-us/.

[22] Yazahmeidi B., Holman C.D.J. (2007). A survey of suppression of public health information by Australian governments. Aust. N. Z. J. Public Health.

[23] McKenzie A., Wale J., McLure K., Hill I., Rumley H., Colbung M., Daniels B., Powell L., Cuesta-Briand B. (2008). Response to ‘A survey of suppression of public health information by Australian governments’. Public Health.

[24] Allen J., Holman C.D., Meslin E.M., Stanley F. (2013). Privacy protectionism and health information: Is there any redress for harms to health?. J. Law Med..

[25] Price S.D., Holman C.D., Sanfilippo F.M., Emery J.D. (2014). Are older Western Australians exposed to potentially inappropriate medications according to the Beers criteria? A 13-year prevalence study. Australas J. Ageing.

[26] Price S.D., Holman C.D., Sanfilippo F.M., Emery J.D. (2014). Association between potentially inappropriate medications from the Beers criteria and the risk of unplanned hospitalization in elderly patients. Ann. Pharmacother..

[27] Einarsdóttir K., Preen D.B., Emery J.D., Holman C.D. (2010). Regular primary care decreases the likelihood of mortality in older people with epilepsy. Med. Care.

[28] Einarsdóttir K., Preen D.B., Emery J.D., Kelman C., Holman C.D. (2010). Regular primary care lowers hospitalisation risk and mortality in seniors with chronic respiratory diseases. J. Gen. Intern. Med..

[29] (2006). National Collaborative Research Infrastructure Strategy Strategic Roadmap. https://www.dese.gov.au/national-research-infrastructure/resources/national-collaborative-research-infrastructure-strategy-strategic-roadmap-2006.

[30] Flack F., Smith M. (2019). The Population Health Research Network—Population data centre profile. Int. J. Pop. Data Sci..

[31] About Health Data Research Network Canada. https://www.hdrn.ca/en/about.

[32] Integrated Data Infrastructure. https://www.stats.govt.nz/integrated-data/integrated-data-infrastructure/.

[33] The National Patient Register. https://econ.au.dk/the-national-centre-for-register-based-research/danish-registers/the-national-patient-register/.

[34] Productivity Commission 2017 Data Availability and Use: Overview & Recommendations, Report No. 82, Canberra. https://www.pc.gov.au/inquiries/completed/data-access/report.

[35] Moore H.C., Guiver T., Woollacott A., De Klerk N., Gidding H.F. (2016). Establishing a process for conducting cross-jurisdictional record linkage in Australia. Aust. N. Z. J. Public Health.

[36] PHRN Annual Review 2019–20. https://www.phrn.org.au/media/82004/phrn-annual-review-2019-2020_digital-v10.pdf.

[37] Young A., Flack F. (2018). Recent trends in the use of linked data in Australia. Aust. Health Rev..

[38] SURE. https://www.saxinstitute.org.au/our-work/sure/.

[39] Impact Stories. https://www.phrn.org.au/for-the-community/impact-stories.

[40] Brotherton J.M.L., Malloy M., Budd A.C., Saville M., Drennan K.T., Gertig D.M. (2015). Effectiveness of less than three doses of quadrivalent human Papillomavirus vaccine against cervical intraepithelial neoplasia when administered using a standard dose spacing schedule: Observational cohort of young women in Australia. Papillomavirus Res..

[41] Clothier H.J., Lee K.J., Sundararajan V., Buttery J.P., Crawford N.W. (2013). Human Papillomavirus vaccine in boys: Background rates of potential adverse events. Med. J. Aust..

[42] Crowe E., Pandeya N., Brotherton J.M.L., Dobson A.J., Kisely S., Lambert S.B., Whiteman D.C. (2014). Effectiveness of quadrivalent human Papillomavirus vaccine for the prevention of cervical abnormalities: Case-control study nested within a population based screening programme in Australia. Br. Med. J..

[43] Population Health Research Network Linked Data in the Fight against COVID-19. https://www.phrn.org.au/media/82108/impact-story-linked-data-in-the-fight-against-covid-19.pdf.

